# Psychosocial Correlates of the Experience of Caregiving Among Caregivers of Patients With Schizophrenia

**DOI:** 10.7759/cureus.58531

**Published:** 2024-04-18

**Authors:** Sanimar S Kochhar, Ashwani K Mishra, Rakesh K Chadda, Mamta Sood, Rachna Bhargava

**Affiliations:** 1 Clinical Psychology, All India Institute of Medical Sciences, New Delhi, New Delhi, IND; 2 Biostatistics, All India Institute of Medical Sciences, New Delhi, New Delhi, IND; 3 Psychiatry, Amrita Hospital, Faridabad, IND; 4 Psychiatry, AIl India Institute of Medical Sciences, New Delhi, New Delhi, IND

**Keywords:** mental illness, coping, family functioning, social support, quality of life, knowledge of illness, caregiver distress, experience of caregiving, caregivers, schizophrenia

## Abstract

Background: Family caregivers provide essential support to their loved ones with schizophrenia with profound outcomes for themselves. The caregiver burden fails to consider the entire caregiving experience, which also incorporates positive aspects of caring. Many potentially significant variables are associated with this.

Aim: To examine the correlates of the experience of caregiving in caregivers of patients with schizophrenia. The specific objectives were to examine the socio-demographic variables of the patients and caregivers, clinical variables of the patient, caregivers’ knowledge of illness, caregivers’ perspectives of family functioning, caregiver coping, their social support, psychological distress, quality of life, and their spirituality, religiosity and personal beliefs and the associations of these variables with the caregivers’ experience of caregiving.

Methods: This cross-sectional observational study was conducted between August 2018 and January 2021 at All India Institute of Medical Sciences, New Delhi, India. One hundred and fifty-eight dyads of patients with schizophrenia and their family caregivers were recruited using purposive sampling. Experience of Caregiving Inventory was used to evaluate the caregiving experience. The caregivers were also assessed on socio-demographics, knowledge of illness, family functioning, coping, social support, general mental health, quality of life, and spiritual, religious, and personal beliefs. Patient socio-demographics and clinical variables were also assessed.

Results: A negative experience of caregiving was reported by caregivers of patients who had higher positive or negative symptoms of schizophrenia. Impaired Communication, Roles, Affective Responsiveness, Affective Involvement, and General Functioning aspects of family functioning were associated with a negative experience of caregiving. Denial/blame and seeking social support as coping were also associated with a negative experience of caregiving. A negative experience of caregiving was significantly positively correlated with greater psychological distress and poorer quality of life. Greater inner peace was associated with a less negative experience of caregiving. Spiritual strength was associated with a more positive experience of caregiving. Knowledge of mental illness and caregiver social support were not significantly associated with the experience of caregiving.

Conclusion: Experience of caregiving is a relevant construct, the understanding of which can help inform caregiver-directed interventions in the future. Specifically, family-based interventions, which include ameliorating patient symptomatology, improving the family environment, strengthening caregivers’ coping strategies, attending to caregiver distress, and encouraging spirituality among caregivers, may lead to a less negative and more positive experience of caregiving; and a better quality of life for caregivers.

## Introduction

Schizophrenia is a chronic, severe, and disabling psychotic illness characterized by disturbances in thought processes, perceptions, emotional responsivity, and social interactions [1]. Schizophrenia affects about 24 million persons or one in 300 persons (0.32%) around the world [2] and is one of the top 15 causes of disability worldwide [3]. About 90% of untreated schizophrenia patients reside in low-to-middle-income countries [4]. The humanistic impact of schizophrenia is massive, affecting the quality of life, causing stigmatization and violence, leading to low rates of employment, marriage, and fertility, and high rates of substance abuse [5].

A caregiver’s role is crucial in this illness. The origins of caregiving behavior can be traced back to anthropology, where the survival of persons with significant impairments is evidence of compassion and moral decency demonstrated in prehistoric societies [6]. Hermanns and Mastel-Smith (2012) identified the essential elements of caring; which include the caregiver’s commitment to the improvement of the care recipient [7]. The emotions identified by them were love, affection, empathy and compassion, satisfaction, and fulfilment in the caregiving role. The most important element was the emotional connection between the caregiver and the care recipient; which characterized the caregiving situation.

Caregiving in schizophrenia can be quite stressful. The common impacts on caregivers include depression, anxiety, and physical issues. Negative experiences of caregiving comprise physical, emotional, and economic impacts; and stigma in caregivers [8]. A very important factor in how caregivers respond is their cognitive appraisal regarding the illness, how they interpret and make sense of the disorder, and how they assess their coping capabilities [9]. Caregiving appraisal is a neutral concept comprising subjective cognitive and affective appraisals of the likely stressors and the efficacy of an individual’s coping strategies [10].

Recently, some positive and beneficial aspects of the caregiving role have come to the forefront, like caregivers acquiring greater sensitivity and strength [11]. The issue with the concept of caregiver ‘burden’ is that it cannot highlight the potential rewarding aspects of caregiving, and that it does not come within a psychological or social theoretical framework that pertains to determinants, mediating factors, and outcomes [12].

The clinical relevance of caregiving in mental illness underscores that the concept of burden considers all negative outcomes in the caregiver’s life to the patient’s illness and does not take into account other factors [12]. Also, it has been shown that the experience of caregiving more accurately predicts caregivers’ psychological well-being rather than burden [9]. 

A study on the experience of caregiving is required because caregiving is now seen to be a complex healthcare activity. Starting from an informal family duty, it is currently a major part of health care [13]. There are very few large sample studies on caregiving in India. Most studies have conducted research taking samples of about 50 to 100 participants. Also, most Indian studies have concentrated on delineating caregiver burden. Caregiving appraisal (as opposed to burden) has been taken up and studied to a limited extent in India [14-17]. Even in the West, the experience of caregiving has been studied infrequently, especially in the area of schizophrenia per se. Regarding Indian studies, these have assessed the association of the experience of caregiving with variables such as patient symptomatology [14-17], coping [14,15,17], social support [14,17], caregiver distress [14,16,17]; and one study that considered familism and family cohesion [17] Out of these studies, one considered only two main variables [15], one compared caregiving in schizophrenia and BPAD [16], and one studied the correlates of caregiver distress [17]. One old study has discussed some variables as correlates of the experience of caregiving [14], but many pertinent variables have not been included in it. Hence, very few studies have been carried out on the knowledge of schizophrenia, positive aspects of caregiving, and spiritual, religious, and personal beliefs in caregivers; and further research is needed in these areas. Thus, we wanted to extend the previous work by including a different combination of variables not studied earlier (including knowledge of illness, positive caregiving experiences, spirituality, religiosity, and personal beliefs) and to consider these aspects; which have not been emphasized in other studies; in the context of quality of life, psychological distress, and social support of the caregivers. Burden captures only a part of the entire caregiving experience, and that too, a negative part; whereas the latter is a much broader construct. Hence, the aim of the present research was to examine the correlates of the experience of caregiving among the caregivers of patients with schizophrenia. Specifically, we examined caregivers’ knowledge of the illness, their perspective of family functioning, coping, social support, psychological distress, quality of life, spirituality, religiosity, and personal beliefs in relation to their experience of caregiving. It was expected that both a negative and positive experience of caregiving would be reported by caregivers; and that it would be associated with the socio-demographics of the patients and caregivers, clinical variables of the patient, caregivers’ knowledge of illness, their perspective of family functioning, their coping, social support, psychological distress, quality of life, and their spiritual, religious and personal beliefs.

## Materials and methods

Setting and participants

This cross-sectional observational study was carried out in the Department of Psychiatry at All India Institute of Medical Sciences (AIIMS), New Delhi. It provides treatment to referred and non-referred patients from different parts of India. Both inpatient and outpatient services are available. The treatment is provided by a team of trained psychiatrists, psychologists, nurses, and other health professionals. The treatment is considerably subsidized. The clientele comprises patients with different psychiatric disorders. Ethical approval for this study was obtained from the Institute Ethics Committee, All India Institute of Medical Sciences (AIIMS), New Delhi (Approval Number: IECPG-204/10.05.2018).

Sample

The sample was drawn from the out-patient services of the Department of Psychiatry using purposive sampling (n=158), with predetermined inclusion and exclusion criteria. Patients with schizophrenia and their caregivers were recruited as dyads.

Inclusion and exclusion criteria

Patients of either gender were included if they had a diagnosis of schizophrenia as per International Statistical Classification of Diseases and Related Health Problems (ICD)-10 criteria of more than one year duration, and were aged between 18-55 years. Caregivers of either gender were included if they were living with the patient for more than one year and were primarily responsible for caring for the patient and supervising their treatment (including medication and hospital visits); could read Hindi (the local language) and comprehend instructions for the assessment and provided informed consent. Patients were excluded if they had comorbid and disabling chronic physical or psychiatric disorders, had substance dependence (except tobacco), or had organic brain syndromes. Caregivers were excluded if they had diagnosed disabling physical or psychiatric disorder or if they did not have regular contact with the patient, and were not living with the patient. Participants with a family member with a diagnosed disabling chronic physical illness or chronic disabling psychiatric disorder staying in the same dwelling unit were also excluded.

Procedure

**Figure 1:**
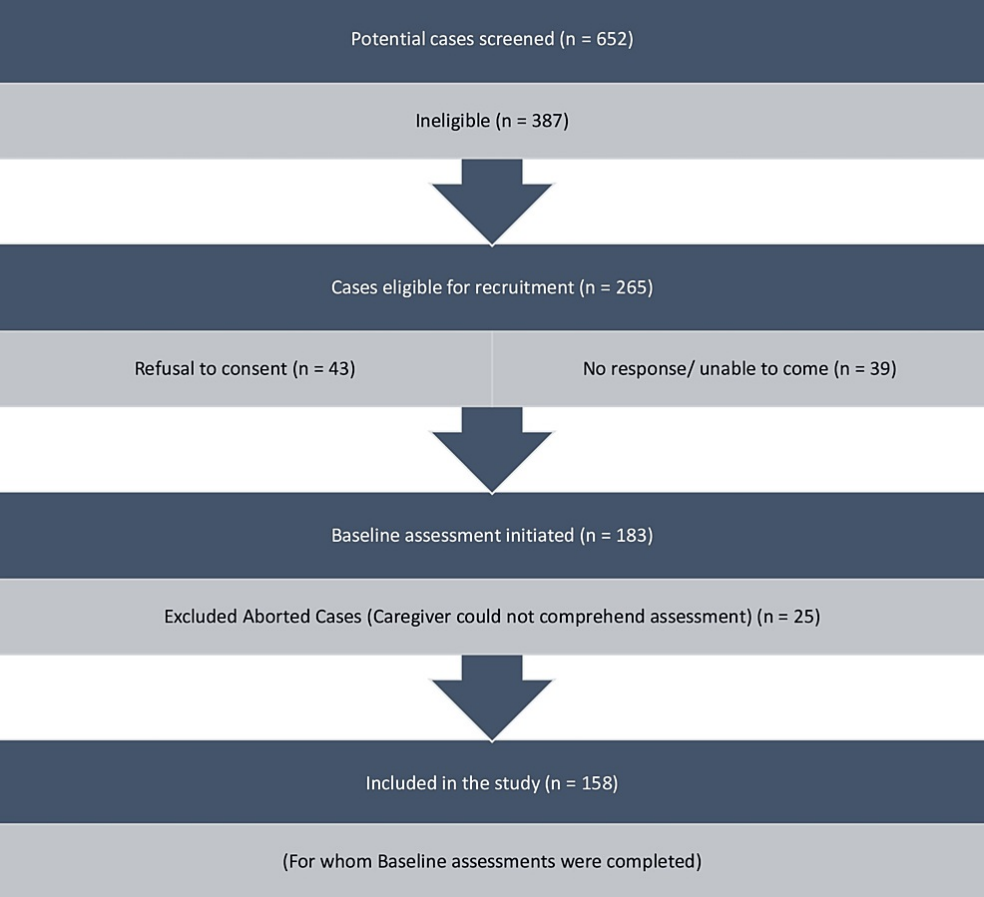
Study recruitment process

The study was commenced after receiving ethical clearance from the Institute Ethics Committee. Existing or new patients suffering from schizophrenia as diagnosed by a consultant psychiatrist, coming to psychiatry outpatient services were screened. Those accompanied by a caregiver and fulfilling the eligibility criteria were taken up for the study. They were explained the nature and purpose of the study and informed consent was sought from both the patient and his/ her primary caregiver. Patients and caregivers who gave their informed consent were then interviewed and were administered the scales. The assessments were completed in one sitting with breaks. Patients were assessed for their demographic (e.g., age, gender, residence) and clinical details (e.g. total duration of illness, total duration of treatment). They were assessed for positive and negative symptoms of schizophrenia using the Scale for the Assessment of Positive Symptoms and Scale for the Assessment of Negative Symptoms respectively. Similarly, the caregivers were assessed for their demographic details. Caregiving experiences were assessed using the Experience of Caregiving Inventory. They were also assessed for knowledge of illness using the Knowledge of Mental Illness scale, family functioning by Family Assessment Device (FAD), coping using the Coping Checklist, social support using the Social Support Questionnaire-Hindi, psychological distress using General Health Questionnaire-12, quality of life using the World Health Organization’s Quality of Life Questionnaire (WHOQOL-Bref), and spirituality, religiosity and personal beliefs using the WHOQOL-SRPB (spiritual, religious and personal beliefs). Figure 1 shows the sample recruitment process. 183 caregivers were eligible and gave consent; however, the assessment was aborted for 25 caregivers who could not comprehend the assessment questionnaires. Therefore, 158 caregivers for whom baseline assessments were completed, were included in the study.

Measures

The Experience of Caregiving Inventory [12] was used to assess the caregiving experience as appraised by caregivers. In this scale, caregiving is conceptualized in a stress-appraisal coping framework. It is a 66-item self-report instrument measuring the experience of caring for a relative with serious mental illness. It consists of 10 independent dimensions in relatives’ appraisal of caregiving: eight negative and two positive. The negative subscales include: difficult behaviors, negative symptoms, stigma, problems with services, effects on the family, loss, dependency, and need for backup. The positive subscales include positive personal experiences and good aspects of the relationship with the patient. The items are rated on a 5-point Likert scale. The negative and positive domains can be summed up to give two measures: total negative score and total positive score. The subscale Cronbach alphas range from 0.74 for dependency to 0.91 for difficult behaviors. This scale was translated into Hindi by three experts and checked for equivalence by three back translations.

The Knowledge of Mental Illness (KMI) Scale [18] assesses the knowledge regarding diagnosis, symptoms, causes, medication, and treatment of mental illness. It consists of five items rated on a 2-point scale. Higher scores represent better knowledge of the illness.

The Family Assessment Device (FAD) [19] was used to assess family functioning. It is a 60-item self-report questionnaire that has its basis in the McMaster model of family functioning. A family member rates how well an item describes their family and each item is scored on a 4-point scale. Higher scores mean poorer family functioning. It has the following seven dimensions: Problem-solving, communication, roles, affective responsiveness, affective involvement, behavioral control, and general family functioning. General functioning assesses overall family functioning. The Cronbach’s alphas in the various domains range from 0.72-0.92. This scale was translated and back-translated for use.

A Coping Checklist [20] was used to assess coping. This 70-item questionnaire covers a wide range of cognitive, behavioral, and emotional responses that are utilized to cope with stress. It has seven subscales, out of which one is problem-focused (Problem-Solving), five are emotion-focused (Distraction-positive, Distraction-negative, Acceptance/Redefinition, Religion/Faith, Denial/Blame), and Social Support which has both problem and emotion-focused elements. The internal consistency of this scale has been seen as adequate (full scale: alpha=0.86).

Social Support Questionnaire-Hindi [21], which is a Hindi adaptation of Pollack and Harris’ (1983) Social Support Questionnaire; was used to assess social support. It consists of 18 items on a 4-point Likert scale. A higher score indicates greater social support. The test-retest reliability of the modified version of the SSQ is high (correlation coefficient= 0.91, p<0.01).

General Health Questionnaire-12 (Hindi Version) [22] was used to assess psychological distress. It is a widely used screening instrument in both clinical and non-clinical settings. A cutoff score of <2 indicates that the caregiver is free from any psychiatric illness.

The WHOQOL-Bref (Hindi Version) [23] was used to assess the quality of life. It is a multidimensional generic instrument. It gives four domain scores: Physical Health, Psychological Health, Social Relationships, and Environment. It has 26 items, scored from 1 to 5. The psychometric properties of this scale are comparable to the full version. It has good discriminant validity, concurrent validity, internal consistency and test-retest reliability. 

The WHOQOL-SRPB (Hindi Version) [24] was used to assess spirituality, religiosity, and personal beliefs. The Hindi version scale consists of 32 items divided into 8 facets, namely, spiritual connection, meaning and purpose in life, experiences of awe and wonder, wholeness and integration, spiritual strength, inner peace, hope and optimism; and faith. Each facet has 4 questions rated on a 5-point Likert scale. The Cronbach alpha ranges from 0.77 to 0.95; and for the complete instrument, it is excellent (0.93).

The patients were assessed using Scale for the Assessment of Positive Symptoms (SAPS), a well-recognized 34-item scale, designed to assess the positive symptoms of schizophrenia patients [25], and Scale for the Assessment of Negative Symptoms (SANS), another commonly used 25-item instrument to assess the negative symptoms of schizophrenia [26].

Statistical analysis

The data were analyzed by the licensed statistical package SPSS 25.0 version software (IBM SPSS Statistics for Windows, Version 25). The data were checked for normalcy using the Kolmogorov-Smirnov test and were summarized using descriptive statistics. Mean and standard deviation and median and inter-quartile range were used for continuous variables, and frequency and percentage were used for the categorical variables. The Spearman rho correlation was used for estimating the relationship between the caregiving experience and the other continuous psychosocial variables. Other parametric and non-parametric tests were used as applicable. The two-sided p<0.05 was considered statistically significant, and for multiple exploratory correlations, correction for multiple comparisons was done using the Bonferroni method. 

Correlations and comparisons (the latter for discontinuous variables) were carried out with the Total Negative Score and Total Positive Score of caregiving experience respectively. The variables used for correlations and comparisons are as follows: Socio-demographics of the patients and caregivers: Patient age, caregiver age, patient gender, caregiver gender, patient marital status, caregiver marital status, religion, patient education, caregiver education, patient occupation, caregiver occupation, area of residence, type of family, and caregiver relationship to the patient and duration of caregiving role. Clinical variables of the patients: Total duration of illness, total duration of treatment, SAPS score (positive symptoms), and SANS score (negative symptoms). Psychosocial variables of the caregiver: Knowledge of mental illness score, Family Functioning domains (problem-solving, communication, roles, affective responsiveness, affective involvement, behavioral control, general functioning), Coping domains (problem-solving, distraction positive, distraction negative, acceptance/redefinition, religion/faith, denial/blame, seeking social support), Social support, Caregiver distress, Quality of life (physical health, psychological health, social relationships, environment), Spiritual, religious and personal beliefs (connectedness, meaning in life, experiences of awe and wonder, wholeness and integration, spiritual strength, inner peace, hope and optimism, and faith). 

## Results

A total of 158 dyads of patients and caregivers were recruited. Among the caregivers, 27.2% (n=43) were siblings, 26.6% (n=42) were fathers, 25.9% (n=41) were mothers, 13.9% (n=22) were spouses, 5.1% (n=8) were children and 1.3% (n=2) were others (sister-in-law). The mean duration of the caregiving role was 7.7 years (± 5.7 years). Table 1 shows the socio-demographic data of the patients and caregivers. The caregivers were more likely to be of a higher mean age, married, educated above higher secondary, and employed as compared to the patients. The dyads primarily came from an urban background, were of Hindu religion, and belonged to nuclear families.

**Table 1:** Socio-demographic characteristics of the patients and caregivers Data shown as mean and SD (Standard Deviation)/ n (%) where applicable.

Socio-Demographics	Patients (n= 158) n (%)	Caregivers (n=158) n (%)
Age (mean ± SD) (Years)	32.7 ± 8.9	45.6 ± 16.0
Gender		
Male	88 (55.7)	85 (53.8)
Female	70 (44.3)	73 (46.2)
Marital Status		
Unmarried	103 (65.2)	41 (25.9)
Married	39 (24.7)	110 (69.6)
Married but currently alone	16 (10.1)	7 (4.5)
Religion		
Hindu	136 (86.1)	Same
Muslim	16 (10.1)	Same
Sikh	6 (3.8)	Same
Education		
Till Middle School	13 (8.2)	7 (4.4)
High School	22 (13.9)	17 (10.8)
Higher Secondary	47 (29.7)	35 (22.2)
Graduate	61 (38.6)	71 (44.9)
PG & Professional	15 (9.6)	28 (17.7)
Occupation		
Professional	7 (4.4)	31 (19.6)
Skilled Workers	10 (6.4)	30 (19.0)
Unskilled/Semi-Skilled	6 (3.8)	12 (7.6)
Unemployed	90 (57.0)	9 (5.7)
Homemaker	22 (13.8)	39 (24.7)
Retired	0 (0)	23 (14.6)
Student	23 (14.6)	14 (8.8)
Area of Residence		
Rural	11 (7.0)	Same
Semi-Urban	35 (22.2)	Same
Urban	112 (70.8)	Same
Type of Family		
Nuclear	102 (64.6)	Same
Joint/Extended	56 (35.4)	Same

Table 2 presents the different scores pertaining to the experience of caregiving and other psychosocial variables such as knowledge about mental illness, family functioning, caregiver coping, social support, psychological distress, quality of life, and spiritual, religious, and personal beliefs. Patient clinical variables are also shown. In caregiving experience, the dependency domain had the highest mean score, followed by negative symptoms and difficult behavior. The good aspects of the relationship outweighed the positive personal experiences while caregiving. In family functioning, the scores on roles and behavioral control were elevated, demonstrating family distress in these areas. Among the coping strategies employed by caregivers, problem-solving and acceptance/redefinition coping scored the highest. The knowledge of illness was low, the social support was moderate, and psychological distress was present in most caregivers; as 63.3% scored above the cut-off of >=2 on the GHQ-12, indicating probable psychological morbidity. The quality of life was highest in physical health and lowest in psychological health. In spiritual, religious, and personal beliefs, hope and optimism scored the highest. The lower part of Table 2 presents the clinical variables of the patients. The mean duration of illness was 9.5 (± 6.3) years, and the mean duration of treatment was 7.9 (± 5.8) years. 34.8% (n=55) of the patients were hospitalized at least once. All the patients received pharmacotherapy, 13.9% (n=22) had received psychotherapy, and 7.5% (n=12) had received electroconvulsive therapy (ECT).

**Table 2:** Scores on psychosocial variables of the caregiver and clinical variables of the patients Data shown as mean and SD (Standard Deviation). SAPS: Scale for Assessment of Positive Symptoms; SANS: Scale for Assessment of Negative Symptoms.

Psychosocial Variables	Mean ± SD
Caregiver	
Caregiving experience	
Negative Domains	
Difficult Behavior	1.7 ± 0.9
Negative Symptoms	2.1 ± 1.0
Stigma	1.3 ± 0.9
Problems with Services	1.1 ± 0.8
Effects on family	1.1 ± 0.8
Need for Back up	1.4 ± 0.7
Dependency	2.4 ± 0.9
Loss	1.5 ± 0.7
Total Negative Score	80.4 ± 31.1
Positive Domains	
Positive Personal Experiences	1.7 ± 0.8
Good Aspects of the Relationship	2.4 ± 0.8
Total Positive Score	27.8 ± 8.7
Knowledge of mental illness score	1.6 ± 1.5
Family functioning	
Problem Solving	1.5 ± 0.5
Communication	1.9 ± 0.5
Roles	2.3 ± 0.5
Affective Responsiveness	2.0 ± 0.7
Affective Involvement	1.9 ± 0.6
Behavioral Control	2.1 ± 0.6
General Functioning	1.7 ± 0.6
Coping	
Problem Focused	
Problem Solving	0.6 ± 0.2
Emotion Focused	
Distraction Positive	0.4 ± 0.2
Distraction Negative	0.1 ± 0.1
Acceptance/Redefinition	0.6 ± 0.2
Religion/Faith	0.4 ± 0.2
Denial/Blame	0.4 ± 0.4
Problem and Emotion Focused	
Seeking Social Support	0.5 ± 0.3
Social Support	51.5 ± 8.2
Psychological Distress	4.4 ± 4.2
Quality of Life	
Physical Health	16.1 ± 2.7
Psychological Health	14.6 ± 2.6
Social Relationships	14.9 ± 2.9
Environment	15.2 ± 2.8
Spiritual, religious & personal beliefs	
Connectedness	3.3 ± 1.1
Meaning in Life	3.7 ± 0.8
Awe and Wonder	3.7 ± 0.7
Wholeness and Integration	3.6 ± 0.8
Spiritual Strength	3.5 ± 1.2
Inner Peace	3.4 ± 0.8
Hope and Optimism	3.8 ± 0.9
Faith	3.6 ± 0.9
Patient Clinical Variables	
SAPS score	19.8 ± 16.9
SANS score	38.3 ± 17.1
Total Duration of Illness (Years)	9.5 ± 6.3
Total Duration of Treatment (Years)	7.9 ± 5.8
Duration of Caregiving Role (Years)	7.7 ± 5.7

Table 3 presents the associations of various socio-demographic variables of the patient and caregiver with the negative and positive experiences of caregiving. After Bonferroni Correction, none of the socio-demographics of the patient and caregiver had any significant association with the experience of caregiving.

**Table 3:** Association of patients' and caregivers' socio-demographics with the experience of caregiving Shown as rho (Spearman correlations) or mean and standard deviation. Parametric and non-parametric tests utilized as applicable. Significance set at p<0.00104 (after Bonferroni correction).

Variable	Total Negative Score mean ± SD	p-value	Total Positive Score mean ± SD	p-value
Patient Age (Years) (Rho)	-0.004	0.580	0.004	0.956
Caregiver Age (Years) (Rho)	-0.120	0.132	-0.025	0.760
Patient Gender				
Male	86.9 ± 27.5	0.004	29.1 ± 8.0	0.038
Female	72.3 ± 33.6		26.2 ± 9.3	
Caregiver Gender				
Male	78.4 ± 29.7	0.390	26.5 ± 8.6	0.039
Female	82.7 ± 32.7		29.3 ± 8.7	
Patient Marital Status				
Never Married	82.8 ± 32.2	0.420	28.1 ± 9.1	0.235
Married	77.9 ± 30.8		28.3 ± 8.4	
Separated	72.4 ± 29.6		27.3 ± 6.7	
Divorced/Widowed	70.1 ± 11.9		21.7 ± 6.6	
Caregiver Marital Status				
Never Married	85.6 ± 31.7	0.319	28.2 ± 9.0	0.234
Married	78.3 ± 29.9		27.4 ± 8.8	
Divorced/Widowed	83.6 ± 45.1		32.4 ± 4.5	
Religion				
Hindu	79.5 ± 31.8	0.384	27.4 ± 8.6	0.096
Muslim	83.7 ± 25.7		32.1 ± 9.3	
Sikh	91.7 ± 29.6		25.8 ± 8.8	
Patient Education				
Illiterate & Primary	68.8 ± 21.9	0.807	27.6 ± 11.8	0.244
Middle	80.5 ± 26.5		28.8 ± 10.2	
Matric	78.6 ± 25.1		29.8 ± 7.7	
Higher Secondary	78.9 ± 31.2		28.3 ± 8.3	
Graduate	81.6 ± 34.2		26.1 ± 9.5	
Post Graduate & Professional	87.1 ± 32.7		30.0 ± 5.5	
Caregiver Education				
Middle	70.9 ± 21.2	0.520	28.6 ± 8.5	0.475
Matric	74.4 ± 33.6		27.4 ± 9.8	
Higher Secondary	83.1 ± 28.3		27.7 ± 8.7	
Graduate	78.9 ± 33.8		26.9 ± 8.8	
Post Graduate & Professional	86.9 ± 27.7		30.2 ± 8.0	
Patient Occupation				
Professional	76.3 ± 24.8	0.172	28.7 ± 9.8	0.348
Skilled Worker	60.6 ± 36.2		29.6 ± 5.9	
Unskilled Worker	84.7 ± 19.9		33.7 ± 8.5	
Unemployed	87.5 ± 30.4		28.3 ± 8.5	
Homemaker	69.2 ± 29.1		25.7 ± 8.8	
Student	70.2 ± 32.6		25.1 ± 8.6	
Business	70.8 ± 34.3		31.0 ± 12.3	
Caregiver Occupation				
Professional	78.0 ± 32.5	0.843	26.0 ± 8.3	0.515
Skilled Worker	73.2 ± 25.1		24.9 ± 8.1	
Unskilled Worker	76.9 ± 26.2		30.1 ± 9.1	
Unemployed	88.6 ± 24.5		30.7 ± 11.6	
Homemaker	83.9 ± 33.6		27.9 ± 8.3	
Retired	78.4 ± 35.4		28.6 ± 8.0	
Student	81.5 ± 37.5		30.8 ± 9.7	
Business/Farmer	86.2 ± 22.4		28.6 ± 9.5	
Area of Residence				
Rural	88.8 ± 30.1	0.428	32.6 ± 7.2	0.162
Semi-Urban	75.6 ± 28.6		27.4 ± 9.6	
Urban	81.1 ± 31.9		27.5 ± 8.5	
Type of Family				
Nuclear	79.0 ± 32.8	0.430	27.1 ± 8.9	0.147
Joint & Extended	82.1 ± 27.9		29.2 ± 8.2	
Caregiver Relationship to Patient				
Mother	74.1 ± 35.7	0.364	28.1 ± 8.6	0.554
Father	81.4 ± 28.2		27.1 ± 8.9	
Brother	82.5 ± 30.0		27.0 ± 7.9	
Sister	95.3 ± 28.9		31.8 ± 8.7	
Spouse	79.2 ± 31.3		26.9 ± 8.9	
Son/Daughter & Other	76.9 ± 26.3		28.0 ± 10.4	

Table 4 presents the relationship of the experience of caregiving (both positive and negative) with caregiver and patient-related variables. Since there were 34 comparisons with these, along with 14 comparisons with socio-demographics (shown in Table 1, plus the caregiver’s relationship to the patient), we applied the Bonferroni correction for p-value at 0.05/48 (~0.00104). We found that none of the patients’ and caregivers’ socio-demographics had a significant association with either the negative or positive experience of caregiving. The duration of the caregiving role also had no significant correlation with the experience of caregiving. In family functioning, the total negative score of the experience of caregiving was significantly positively correlated with communication, roles, affective responsiveness, affective involvement, and general functioning. Denial/blame and seeking social support as coping strategies were significantly positively correlated with a negative experience of caregiving. A negative experience of caregiving was associated with psychological distress as measured by the GHQ-12 in the caregivers. A higher negative experience of caregiving was also associated with poorer quality of life in all domains. Greater Inner peace in spiritual, religious, and personal beliefs was associated with a lower negative experience of caregiving. Higher spiritual strength was associated with a more positive experience of caregiving. Higher SAPS scores and higher SANS scores were associated with a higher negative experience of caregiving. Knowledge of mental illness and caregiver social support were not significantly associated with either a positive or negative experience of caregiving.

**Table 4:** Correlations of the experience of caregiving with other psychosocial variables of the caregiver Data shown as rho (Spearman correlations). *Significant at p<0.00104 (after Bonferroni correction); SAPS: Scale for Assessment of Positive Symptoms; SANS: Scale for Assessment of Negative Symptoms.

Psychosocial Variables	Experience of Caregiving Total Negative Score (rho)	p-value	Experience of Caregiving Total Positive Score (rho)	p-value
Caregiver				
Knowledge of mental illness	-0.018	0.823	0.038	0.633
Family functioning				
Problem Solving	0.221	0.005	-0.116	0.146
Communication	0.307	<0.001*	0.034	0.670
Roles	0.393	<0.001*	-0.038	0.638
Affective Responsiveness	0.287	<0.001*	-0.079	0.322
Affective Involvement	0.309	<0.001*	0.047	0.554
Behavioral Control	0.164	0.039	-0.116	0.147
General Functioning	0.402	<0.001*	-0.054	0.499
Coping				
Problem Focused				
Problem Solving	0.160	0.044	0.228	0.004
Emotion Focused				
Distraction Positive	0.051	0.522	0.147	0.066
Distraction Negative	0.193	0.015	0.028	0.722
Acceptance/Redefinition	0.091	0.256	0.225	0.005
Religion/Faith	0.106	0.185	0.106	0.184
Denial/Blame	0.352	<0.001*	0.141	0.078
Problem and Emotion Focused				
Seeking Social Support	0.269	0.001*	0.216	0.006
Social Support	-0.240	0.002	0.108	0.178
Psychological Distress	0.528	<0.001*	0.179	0.024
Quality of Life				
Physical Health	-0.340	<0.001*	0.001	0.988
Psychological Health	-0.425	<0.001*	0.125	0.118
Social Relationships	-0.297	<0.001*	0.155	0.052
Environment	-0.297	<0.001*	0.176	0.027
Spiritual, Religious & Personal Beliefs				
Connectedness	-0.033	0.677	0.208	0.009
Meaning in Life	-0.109	0.172	0.175	0.027
Awe and Wonder	-0.039	0.631	0.174	0.028
Wholeness and Integration	-0.151	0.059	0.156	0.050
Spiritual Strength	-0.016	0.845	0.261	0.001*
Inner Peace	-0.304	<0.001*	0.105	0.188
Hope and Optimism	-0.096	0.231	0.189	0.018
Faith	-0.160	0.045	0.173	0.030
Patient Clinical Variables				
Total duration of illness	0.027	0.735	0.067	0.406
Total duration of treatment	0.070	0.380	0.098	0.219
SAPS score	0.458	<0.001*	0.211	0.008
SANS score	0.269	0.001*	0.107	0.181
Duration of Caregiving Role	-0.004	0.965	-0.019	0.809

## Discussion

This study aimed to examine the correlates of the experience of caregiving among caregivers of patients with schizophrenia. In India, most patients with schizophrenia are residing with their families [27]. The experience of caregiving can be exhausting, but, at the same time, enriching for the caregivers, and Indian society can utilize this commitment of the caregivers [27]. Thus, the caregiving experiences of the caregivers are of value. The present scores on negative experiences of caregiving were somewhat higher than those reported by Aggarwal et al., 2009 [14] and Martens and Addington, 2001 [9], but lower than those reported by Doval et al., 2018 [15] and Grover et al., 2012 [16]. The scores on negative experiences were quite close to the baseline scores of Jorge et al., 2019 [28] among caregivers of patients of first-episode psychosis. The positive experiences of caregiving were higher than those reported by most studies [9,14-16] but lower than those reported by Jorge et al. 2019 [28] in caregivers of patients with first-episode psychosis. In the domain scores, scores were highest on dependency, followed by negative symptoms and difficult behaviors in our study. Similar results were seen in [14-16]. These findings may reflect the symptomatology and the fact that the patients in this study were mostly young, unemployed, and single. The good aspects of the patient-caregiver relationship exceeded the positive personal experiences in our study; and this is also supported by other researches [14,15].

Nonetheless, the present study adds to the literature on the extent of positive and negative experiences of caregiving in patients with schizophrenia. The differences across the studies may be attributable to the differences in samples, duration of caregiving role, familial circumstances, and cultural factors. The relevant factors in our setting pertained to having a sample of outpatients with chronic but relatively stable illness of mild to moderate severity; with caregivers having a greater level of education and being economically better off compared to other areas in the country. Also, the sample was from a tertiary care hospital with accessible care where they hope for the improvement of their patient; as well as coming from a collectivist culture that places value on family obligations for caring. Moreover, the duration of illness and caregiving were not limited, which may lead to adjusting to the illness over its course; and caregivers without chronic disabling conditions were included, providing a sample closer to real life.

The study reveals several correlates of the experiences of caregiving. After the Bonferroni correction, we did not find significant correlations between the socio-demographics of the patient and the caregivers. This is similar to Grover et al., [16]. Our results are at variance with Aggarwal et al. [14], where patient and caregiver education were associated with a positive experience of caregiving; and Doval et al. [15], where caregiver education, family income, and urban residence were associated with the same. We found that negative experiences of caregiving were correlated with the severity of symptoms of schizophrenia in the patients. Previous literature also suggests negative aspects of caregiving to be associated with the severity of psychopathology in patients with schizophrenia [14,15]. Greater manifestations of symptoms of schizophrenia are likely to have a greater effect on the family members and caregivers. One could assume that manifestations like hallucinations, delusions, apathy, and avolition result in a huge stressful impact on the caregivers.

The present study found negative experiences of caregiving to be significantly associated with several aspects of impaired family functioning like communication, roles, affective responsiveness, affective involvement, and general functioning. We cannot infer the directionality of the relationship based on this association, though we could comment that certain aspects of the family environment are related to the experience of caregiving. The plausible explanations could be that caregiving is stressful, which affects family functioning, or altered and distressed family functioning may result in a negative caregiving appraisal; or there can be common underlying factors (like personality) that alter the appraisal of caregiving and family functioning. We did find denial/blame or seeking social support as coping to be significantly associated with a negative experience of caregiving. Aggarwal et al. [14] and Doval et al. [15] also report these to be important coping methods; although the former reported an association of seeking social support with a positive experience of caregiving, which our study did not replicate after the Bonferroni correction.

Our findings are in agreement with most previous research [9,14,16,17,28] that a negative experience of caregiving is associated with caregiver distress and psychological morbidity. In our study, a large number (63.3%) of the caregivers were seen to have probable psychological morbidity. This figure is much higher than those found in other Indian studies (24% in Aggarwal et al. [14], 41.4% in Grover et al. [16] and 48% in Hegde et al. [17]. The probable explanations for this could be that first, caregivers in the capital city of India are stressed due to many other factors as well; and second, this being a study from a tertiary care hospital, many difficult-to-treat cases with their stressed caregivers are more likely to come here. Expectedly, a greater negative appraisal of the caregiving experience was also associated with a poorer quality of life. Previous literature also suggests schizophrenia to be associated with a poorer quality of life among caregivers [29]. The greatest negative impact was on the psychological quality of life of the caregivers. The analysis delves into the association of the different aspects of caregiving with the spiritual, religious, and personal beliefs of the caregivers. A negative experience of caregiving was associated with lower inner peace among the caregivers, while a higher spiritual strength was associated with a positive caregiving experience. Possibly, those who have inner peace are less likely to be affected negatively by the circumstances encompassing the caregiving role. A corollary to that is individuals experiencing higher spiritual strength may appraise the caregiving role as an important epoch in one’s life, and have a positive appraisal of the caregiving role.

The findings of the paper have several implications. Caregivers are very important stakeholders for the well-being of their patients, and, therefore, should be treated as equal partners in care. Their voices should be heard, and their concerns given due attention. They appraise caregiving in both a negative and a positive light, and this should be acknowledged. Firstly, ameliorating patient symptomatology through a regular and effective medication regime with proper adherence should be ensured; to reduce the negative experience of caregiving. Second, family-based interventions should be considered for caregivers; as they have a positive impact on both patient and caregiver outcomes. Improving the family environment through interventions that emphasize proper communication, demarcation of each family member’s role, responding appropriately to each member’s needs and interests, and improving general family functioning will go a long way in reducing the negative experience of caregiving in caregivers. Third, caregiver coping can be strengthened by promoting adaptive coping strategies and reducing maladaptive emotion-focused strategies such as denial and blaming others. There should also be a constructive use of social support, and caregivers should be given access to support groups to build their social networks. Fourth, ‘Caring for the caregivers’ is a very relevant cause; as caregiver stress can negatively impact their ability to care. Thus, caregivers’ psychological issues should be addressed adequately by professionals; to reduce their psychological distress. Lastly, encouraging spirituality among caregivers may lead to an improvement in their inner peace; and bolster their spiritual strength; specifically, the latter may increase the positive experience of caregiving. Implementing suggestions in all these areas may lead to a less negative experience of caregiving and ultimately, a better quality of life for the caregivers.

Although not a part of the study findings, research on the experience of caregiving, especially the positive aspects of caregiving, ought to be conducted to a larger extent. Also, proper psychoeducation may increase the knowledge of illness and result in better management of the illness and may improve the caregiving experience in the future. In terms of caregiver psychoeducation programs, a recently published RCT of family psychoeducation demonstrated that for the patients, it significantly decreased the risk of relapse, and for caregivers, it decreased burden, depression, and increased knowledge of illness [30]. Such programs could be routinely carried out in other settings, including ours. 

The strength of the study lies in using a fairly adequate sample and assessing the experience of caregiving, which is a currently preferred construct; and its relationship with several relevant variables, some of which are less studied, using some culture and region-specific tools. The limitations of the study are that only correlational analysis could be done and no validation process was carried out for the scales translated into Hindi language. A control group to analyze the differences in caregiving was not included. Only one family member (the main caregiver) was assessed using the Family Assessment Device. The patients’ compliance with medication was not assessed and could have been another variable to consider for the associations. Furthermore, the sample was purposive and limited to one location. Generalizations should be hence drawn with caution. Further research can be conducted using a control group; in order to examine how the experience of caregiving differs in caregivers of other medical conditions and caregivers of other psychiatric disorders. A qualitative appraisal of caregivers can be done in order to get rich data on their lived experiences of living and caring for a schizophrenia patient. A longer follow-up can be conducted to accurately measure the changes in caregiving experience as well as its correlates over time; to move a step closer towards establishing causality. The assessment of family functioning could be carried out on all family members to get an idea of how the family functions as a unit as the perspectives of different family members could be different.

## Conclusions

To conclude, the study elaborates on the positive and negative aspects of caregiving among caregivers of patients with schizophrenia. Several other correlates, such as family functioning, quality of life, coping, caregiver distress, and others were also studied as a part of the research. The primary findings of this study were that a greater level of positive and negative symptomatology in patients, impaired family functioning in various domains, and a greater use of maladaptive coping strategies were associated with a more negative experience of caregiving; which was associated with a greater psychological distress in caregivers and a poorer quality of life in all domains. It was also associated with decreased inner peace among caregivers; whereas improving the spiritual strength of caregivers was associated with a more positive experience of caregiving. The knowledge of mental illness and the social support available to caregivers were not significantly associated with the caregiving experience. Comprehensively assessing the experience of caregiving in caregivers is the first step and the foundation for the future development of caregiver and family-based interventions as well as designing policy recommendations for assisting caregivers in their role and responsibility.
